# Evidence of static quenching in the photoredox activity of perylene diimide radical anions: implications for consecutive photoinduced electron transfer photocatalysis

**DOI:** 10.1039/d6sc02462a

**Published:** 2026-05-22

**Authors:** Estefanía Sucre-Rosales, Daniel H. Cruz Neto, Eric Vauthey

**Affiliations:** a Department of Physical Chemistry, University of Geneva 30 Quai Ernest-Ansermet CH-1211 Geneva 4 Switzerland eric.vauthey@unige.ch

## Abstract

The radical anions of perylene diimides (PDI˙^−^*) are increasingly used photocatalysts in consecutive photoinduced electron transfer (conPET) for aryl-halide reduction. Despite this, the mechanism behind this reduction by PDI˙^−^* remains unclear since no dynamic quenching was observed for aryl halides. Here, we combine stationary and ultrafast transient absorption spectroscopy from the UV to the near IR to reinvestigate the photoreactivity of PDI˙^−^ in the presence of bromoacetophenone (BAP). Independently of the method used to generate PDI˙^−^, the solvent, and the excitation wavelength, a prompt decrease of the excited-state population without any change in lifetime is observed upon addition of BAP. This effect coincides with a similar decrease of the stationary fluorescence intensity of PDI˙^−^, pointing to the occurrence of static quenching by BAP. As no photoproduct is detected, this static quenching is associated with a sub-100 fs photoinduced electron transfer, followed by ultrafast recombination by back electron transfer. It does not involve any pre-association of the reactants in the ground state but is simply due to the significant probability of a BAP molecule to be at an adequate distance and orientation relative to PDI˙^−^* for electron transfer to occur without diffusion. Using molecular dynamics simulations, we show that such condition is easily fulfilled at high quencher concentrations. Despite this, the overall quantum yield of the conPET mechanism is vanishingly small, because of several fundamental limitations related to the two photoinduced electron transfer steps and to the use of organic radical ions as photocatalysts.

## Abstract 2

Photoinduced ET between PDI˙^−^ and BAP occurs through static quenching only, and recombination of the ensuing product is too fast for BAP˙^−^ to react further. Therefore, static quenching is detrimental to the efficiency of the conPET mechanism.

## Graphical abstract

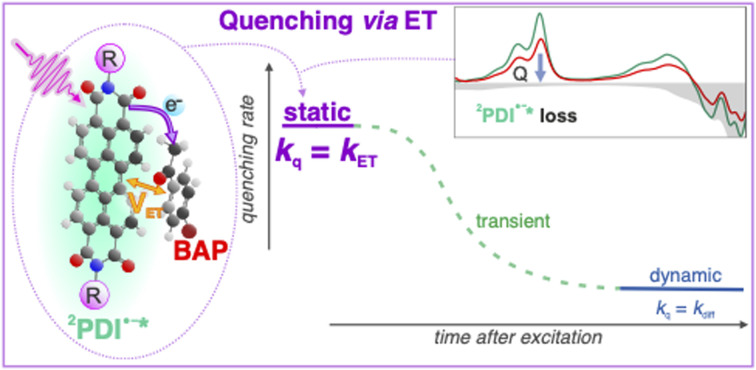

## 1 Introduction: the conPET mechanism and the controversies

Aryl halides, Ar–X, are among the most widely used electrophiles in organic synthesis, serving as key substrates in cross-coupling chemistry, radical transformations, and late-stage functionalisation.^1–6^ However, their reduction is thermodynamically challenging because Ar–X bonds are strong and their corresponding reduction potentials are typically very negative. This makes their activation by visible-light photocatalysis difficult, especially under mild conditions and without the use of transition metals. Consequently, identifying photocatalytic strategies capable of reducing aryl halides is both synthetically valuable and mechanistically significant, motivating continued efforts to understand and optimize such processes.

**Fig. 1:**
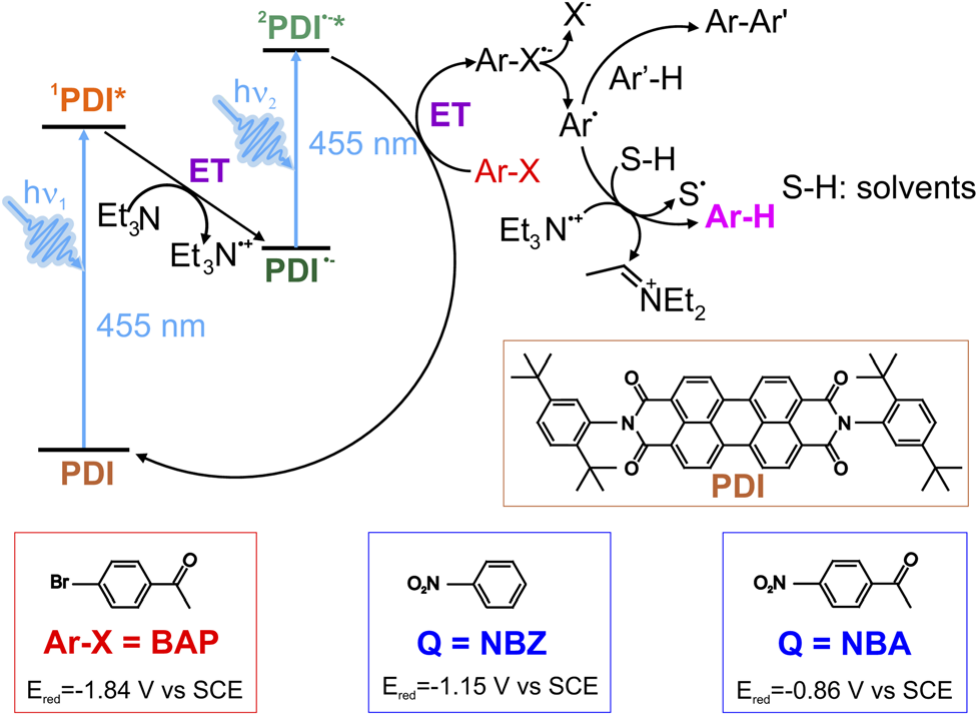
Consecutive photoinduced electron transfer (conPET) mechanism, as originally reported in ref. 7. Perylene diimide derivative and acceptors used in the present work.

The reduction of aryl halides using the radical anion of a perylene diimide, PDI˙^−^, as a photocatalyst was first reported in 2014.^7^ The authors observed that 4-bromoacetophenone (BAP) is reduced to acetophenone (AP) with an 82% yield after four hours of continuous 455 nm irradiation of PDI with triethylamine (TEA) in dimethylformamide (DMF) under an inert atmosphere. Based on the observation of the formation and consumption of PDI˙^−^ by stationary absorption spectroscopy, the authors proposed the so-called “consecutive photo-induced electron transfer” (conPET) mechanism, which, as depicted in Fig. 1, involves two photoexcitation steps in contrast to the previously reported one-photon mechanisms.^4,8–14^ According to this scheme, PDI is first excited to its S_1_ state before being reduced by the sacrificial donor TEA to yield the radical anion PDI˙^−^. In the second photoexcitation step, PDI˙^−^ is brought to an upper doublet excited state, D_*n*>1_, followed by internal conversion to D_1_. Subsequently, PDI˙^−^* undergoes electron transfer with Ar–X to generate its radical anion, which reacts further to eventually produce Ar–H.

This report not only led to more studies of the conPET mechanism with other organic photocatalysts,^15–19^ but it also raised concerns regarding its feasibility. It was first suggested that diffusional quenching would be unlikely because of the very short, 145 ps,^20^ excited-state lifetime of PDI˙^−^, but that pre-association between PDI˙^−^ and Ar–X could be involved.^13^ Later on, a study by Ceroni and co-workers evidenced several issues, which directly challenge the feasibility of this mechanism.^21^ First, the efficiency of the second photoexcitation step was questioned, since PDI˙^−^ absorbs only poorly at 455 nm, with an extinction coefficient approximately 9 times smaller than that of the neutral PDI. Second, it was found that the buildup of the final product starts only once all PDI˙^−^ species are consumed. It was thus concluded that the actual photocatalyst is not PDI˙^−^ itself but rather an unidentified photodegradation product of this anion.^21^

More recently, Zeman *et al.* investigated the photoinduced ET from chemically-generated PDI˙^−^ to several aryl halides and other acceptors in DMF using ultrafast transient absorption (TA) spectroscopy.^22^ The authors found that PDI˙^−^* is quenched with a rate constant close to the diffusion limit by acceptors with reduction potentials more positive than −1.7 V *vs.* SCE, which include halogenated benzaldehydes and nitrobenzene. An oxidation potential of −1.87 V *vs.* SCE was estimated for PDI˙^−^*, rendering the reduction of aryl halides such as BAP (*E*_red_ between −1.84 and −1.89 V *vs.* SCE) inefficient. This was confirmed by the absence of any observable dynamic quenching by BAP as well as other aryl halides like 4-chloroacetophenone and iodobenzene. Concerning the feasibility of the conPET with BAP as originally reported (Fig. 1),^7^ the authors proposed that (i) photoinduced ET may occur but with an efficiency below the detection limit of their experiment (<1%); or (ii) a thermally activated ET could be involved, in agreement with the detection of a slow dark reaction. However, the latter process was only observed with 3- and 4-iodobenzene, but not with BAP. The key question is, therefore, whether PDI˙^−^* is reactive and long-lived enough to reduce these weak aryl-halide acceptors under the conditions reported in the original study.^7^

Interestingly, the TA data presented by Zeman *et al.* exhibit an almost ubiquitous prompt reduction of the initial intensity of the excited-state absorption (ESA) band of PDI˙^−^ in the presence of the quencher, despite maintaining the same anion concentration in all experiments.^22^ Such loss of the initial PDI˙^−^* population, not addressed by the authors, is visible with both the acceptors undergoing dynamic quenching and those reported to not quench PDI˙^−^*, namely iodobenzene and BAP.

Here, we report our investigation of the photoreactivity of PDI˙^−^ in the presence of BAP, focussing on the origin of this prompt decrease of the PDI˙^−^* population. For this, PDI˙^−^ was first prepared chemically and its excited-state dynamics in the presence of BAP was studied by stationary absorption and fluorescence spectroscopy as well as TA spectroscopy. In a second stage, the photoreactivity of PDI˙^−^ generated photochemically *via* two different approaches was investigated using pump–pump–probe spectroscopy.

We show that the prompt loss of the PDI˙^−^* population in the presence of BAP is genuine. This effect, together with the concurrent prompt decrease of the ground-state bleach signal, is explained in terms of static ET quenching by BAP, followed by ultrafast recombination by back ET to PDI˙^−^ + BAP. Contrary to what is often assumed, such static quenching does not need any pre-association of the reactants but is an ubiquitous process resulting from the presence of reactant pairs with an adequate distance/orientation for ET to occur without significant diffusional motion.^23–27^ Such pairs are always present at sufficiently large quencher concentrations, typically above 0.3–0.5 M. Our molecular dynamics simulations indicate that this is also the case for PDI˙^−^ and BAP. Occurrence of static quenching and absence of dynamic quenching with BAP are explained by the large electronic coupling, *V*_ET_, that can be achieved in these reactant pairs and which compensates for the absence of a significant driving force. Despite ET being operative with BAP, back ET of the quenching product is ultrafast because of the large electronic coupling, *V*_BET_, and of its moderate driving force, which corresponds to the barrierless regime in terms of Marcus theory, rather than to the inverted region. Because of this, the radical anion of BAP is so short-lived that its reaction toward AP is extremely inefficient. In principle, this behaviour is not restricted to PDI˙^−^ but is expected for organic open-shell radicals with a short-lived excited state, limiting their efficiency as photoredox catalysts.

These results, together with the different photoreactivities in acetonitrile and DMF, support the previous hypotheses that PDI˙^−^* does not act as the catalyst in the formation of AP.

## 2 Results

### 2.1 Fluorescence quenching of PDI˙^−^ with 4-bromoacetophenone (BAP)

PDI˙^−^ was first prepared as described by Zeman *et al.*,^22^*i.e.*, *via* chemical reduction with tetrakis(dimethylamino)-ethylene (TDAE) under an inert atmosphere in DMF in the presence of one of the acceptors shown in Fig. 1. Both nitrobenzene (NBZ, *E*_red_ = −1.15 V *vs.* SCE) and 4-nitrobenzaldehyde (NBA, *E*_red_ = −0.86 V *vs.* SCE) were reported to dynamically quench PDI˙^−^* with Stern–Volmer constants, *k*_q_, of 1.10 × 10^10^ and 1.22 × 10^10^ M^−1^ s^−1^, respectively.^22^

**Fig. 2:**
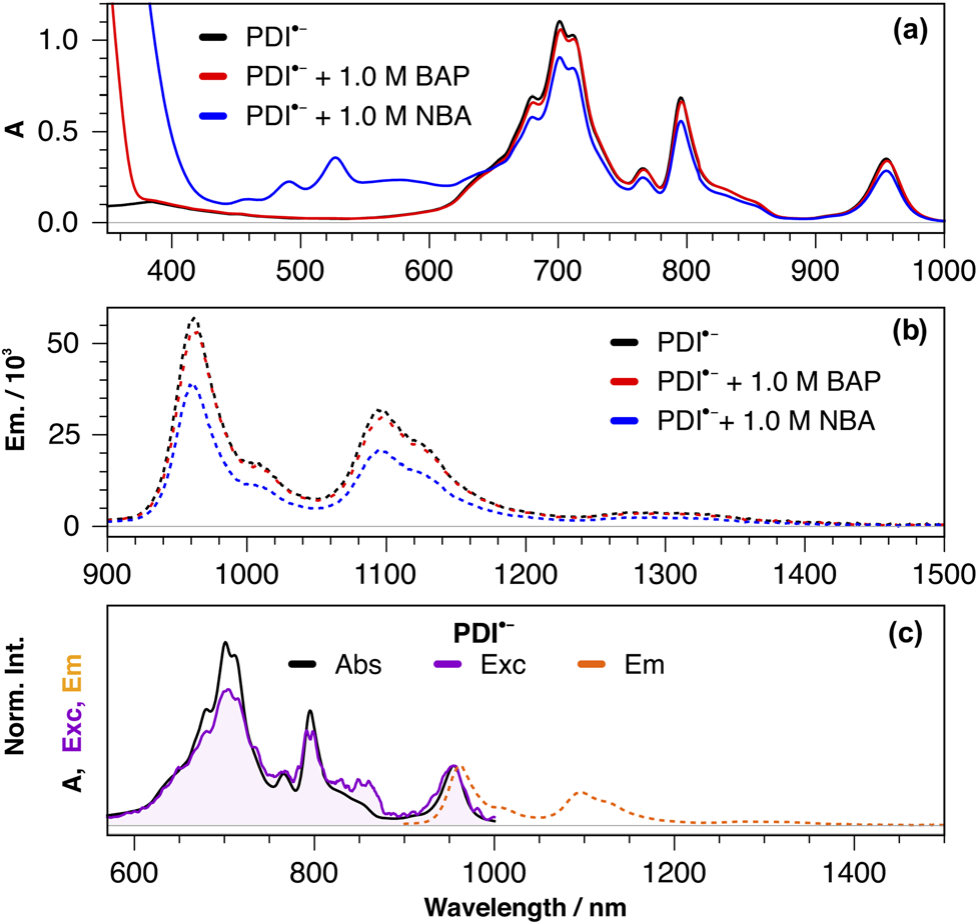
(a) Stationary electronic absorption (a) and emission (b) spectra of PDI˙^−^ in DMF with and without quencher. (c) Comparison of the stationary electronic absorption and fluorescence excitation spectra (measured at 960 nm) and emission spectrum (800 nm excitation).

To avoid any dilution effects, the PDI˙^−^ concentration was kept constant throughout the measurements. As illustrated in Fig. 2a and S1, the stationary electronic absorption spectrum of PDI˙^−^ in the 600–1000 nm region remains unchanged in the presence of BAP and NBZ up to 1 M. Consequently, the formation of a ground-state complex with PDI˙^−^ is improbable. By contrast, in the presence of the stronger acceptor NBA, the spectrum exhibits the characteristic band of the neutral PDI between 400 and 500 nm, as well as a broad feature around 600 nm. The presence of PDI points to a thermal ET and an equilibrium between PDI˙^−^ + NBA and PDI + NBA˙^−^. The broad 600 nm band is consistent with the absorption spectrum of NBA˙^−^ in sulfolane reported in the literature.^28^

Similarly to the radical anion of the parent napthalenediimide rylene,^29,30^ PDI˙^−^ exhibits distinct fluorescence (Fig. 2b). This emission is in the near IR, between 900 and 1400 nm, explaining why it was never reported previously, to the best of our knowledge. The spectrum is characterised by a marked vibronic structure and a small Stokes shift, with the 0–0 emission band overlapping almost entirely with the 0–0 D_1_ ← D_0_ absorption band. Organic radical anions and cations are generally non-fluorescent, mostly because of their very short excited-state lifetime, a few ps or less,^31–43^ due to a small D_1_–D_0_ gap and/or the presence of D_1_–D_0_ conical intersections.^38,44,45,45,46^ Consequently, considering the comparatively long excited-state lifetime of PDI˙^−^ and NDI˙^−^ of about 140 ps,^20^ and the relatively large oscillator strength of the D_1_ ← D_0_ transition, detection of fluorescence from these two radicals should not be surprising. The authenticity of the D_1_ → D_0_ emission of PDI˙^−^ is confirmed by the good match of the fluorescence excitation and absorption spectra, as illustrated in Fig. 2c.

Upon addition of 1.0 M BAP and NBZ, the stationary fluorescence intensity of PDI˙^−^ decreases by about 7% and 33%, respectively (Fig. 2b) These results are direct evidence that BAP quenches, although weakly, PDI˙^−^*.

### 2.2 Revisiting the quenching of chemically generated PDI˙^−^: evidence of static quenching in the presence of aryl halides

**Fig. 3:**
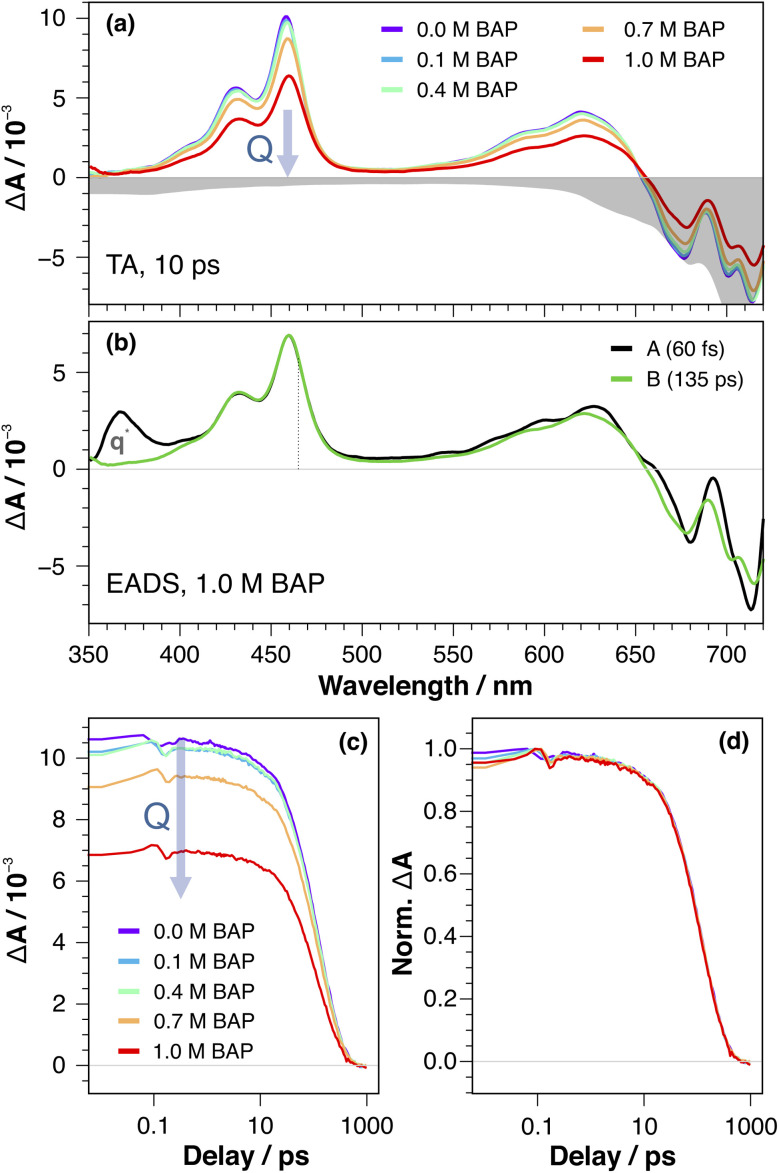
(a) Transient absorption spectra measured 10 ps after 960 nm excitation of PDI˙^−^ with increasing amounts of BAP in DMF. The negative of the stationary absorption spectrum of PDI˙^−^ is shown for comparison. (b) Evolution-associated difference absorption spectra and time constants obtained from a global analysis of the transient absorption data recorded with PDI˙^−^ and 1.0 M BAP, assuming a series to two successive exponential steps (A → B →). The “q*” band is due to the multiphoton excitation of BAP. (c) Time profiles of the transient absorption at 465 nm due to PDI˙^−^* (dotted vertical line in panel b) with increasing amounts of BAP. (d) Same as (c) but after intensity normalisation.

The origin of the fluorescence quenching of PDI˙^−^* was further investigated using TA under the same conditions as reported by Zeman *et al.*^22^ Excitation of PDI˙^−^ was carried out at 800 and 960 nm without and with quencher, keeping both the concentration of PDI˙^−^ and the excitation irradiance constant. All TA data were analysed globally assuming a series of successive exponential steps to obtain evolution-associated difference absorption spectra (EADS) and time constants.^47,48^ As illustrated in Fig. 3 and S4–S6, the TA spectra measured without quencher are dominated by two excited-state absorption (ESA) bands in the 400–470 and 550–650 nm regions as well by negative bands at longer wavelengths, which can be assigned to the ground-state bleach (GSB) and to the D_1_ → D_0_ stimulated emission (SE). Upon 960 nm excitation in the 0–0 D_1_ ← D_0_ band, very little dynamics are observed except for the concurrent decay of all bands with a ∼140 ps time constant (Fig. S5 and S6), in agreement with the PDI˙^−^* lifetime reported previously.^20^ Upon excitation at 800 nm, some early spectral dynamics, mostly band narrowing, which can be assigned to vibrational relaxation of the excess excitation energy,^49^ can additionally be observed (Fig. S4, S8 and S12). Upon addition of BAP, the TA spectra exhibit the same spectral features. Additionally, a short-lived (<100 fs) band around 365 nm due to direct multiphoton excitation of BAP can be observed at high quencher concentrations (Fig. S13). At 0.4 M BAP, all TA bands have a smaller initial amplitude than without quencher. This effect becomes more pronounced as the BAP concentration is increased further, independently of the excitation wavelength. Apart from this, all bands decay with the same ∼140 ps time constant, independently of BAP concentration, as illustrated in Fig. 3c and d. These results confirm the absence of dynamic quenching of PDI˙^−^* by BAP reported earlier.^22^ Additionally, the prompt loss of the ESA band intensity at high quencher concentrations, visible in the TA data presented in ref. 22 but not discussed, is also reproduced here.

Similar measurements were repeated with NBZ and NBA, which were reported to dynamically quench PDI˙^−^*.^22^ The resulting TA spectra are qualitatively similar to those measured with BAP, and also exhibit a prompt reduction of the band intensity at high quencher concentrations (Fig. S8, S10 and S12). However, contrary to BAP, a markedly faster decay of the TA bands is observed. For example, the lifetime of PDI˙^−^* decreases down to 68 ps in the presence of 0.3 M NBZ. Stern–Volmer analysis of the decay time *vs.* quencher concentration yields quenching rate constants close to the diffusion limit in DMF (Fig. S9 and S11), as reported earlier.^22^ As for BAP, no transient band that could be assigned to the ET product, *i.e.*, neutral PDI and the radical anion of the quencher, could be detected. Furthermore, the decay of the GSB band occurs concurrently with that of the ESA bands. This indicates that the decay of the product by back ET is faster than the quenching itself.

Given the occurrence of diffusion-controlled dynamic quenching of PDI˙^−^* by NBZ and NBA, static quenching is necessarily operative at high concentrations,^23–27^ accounting for the prompt initial decay of the ESA bands observed with these two acceptors. Therefore, the prompt ESA decay measured with BAP, which coincides with a decrease of the stationary fluorescence intensity, can also be attributed to static quenching. The reasons why BAP undergo static but not dynamic quenching are discussed below.

### 2.3 Static quenching of photogenerated PDI˙^−^: dimethylformamide *vs.* acetonitrile

We then turned to photoproduction of PDI˙^−^ to investigate its excited-state quenching dynamics under conditions similar to those required for the conPET mechanism, not only in DMF but also in acetonitrile (ACN). Surprisingly, ACN was not used in the original conPET report,^7^ although it is the solvent of choice for photoinduced ET processes.

In a first stage, PDI˙^−^ was produced upon continuous irradiation of PDI at 450 nm in the presence of 100 mM TEA. In DMF, a stationary concentration of PDI˙^−^ could be achieved after about 40 minutes of irradiation (Fig. S2). Surprisingly, such photoaccumulation of PDI˙^−^ was not successful in ACN (Fig. S3). Therefore, only DMF was investigated with this approach. For ACN, another strategy was used, as discussed below.

#### 2.3.1 Photoaccumulation of PDI˙^−^ in DMF

**Fig. 4:**
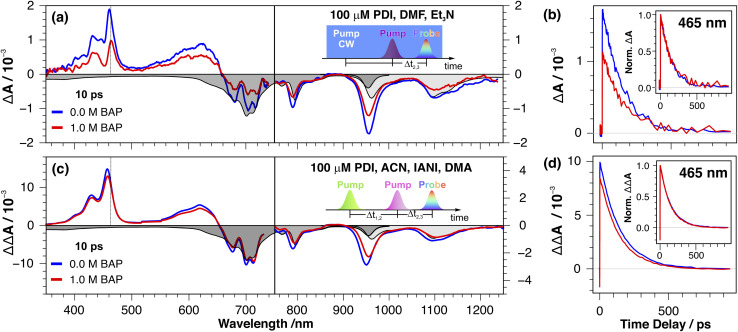
(a) Transient absorption spectra measured without or with 1 M BAP, 10 ps after 800 nm (Vis probing) or 700 nm (NIR probing) excitation of PDI˙^−^ generated upon continuous 450 nm irradiation of deaerated DMF solutions of PDI with 0.1 M TEA. The stationary absorption and stimulated emission spectra of PDI˙^−^ are shown in the shaded gray area for comparison. (b) Time profiles and intensity-normalised time profiles (inset) of the transient absorption at 465 nm due to PDI˙^−^* (dotted vertical line in panel a). (c) Pump–pump–probe transient absorption spectra measured without or with 1 M BAP 10 ps after 800 nm (Vis probing) or 700 nm (NIR probing) excitation of PDI˙^−^ in deaerated acetonitrile, 2 µs after its generation by pulsed excitation at 532 nm of PDI with 1 M iodoanisole (IANI) and 10 mM *N*,*N*-dimethylaniline (DMA). (d) Time profiles and normalised time profiles of the transient absorption at 465 nm due to PDI˙^−^*.

TA measurements in DMF were performed after about 40 min continuous illumination at 450 nm to reach a sufficient stationary concentration of PDI˙^−^. The latter was then excited with a fs pulse at 800 or 700 nm for probing in the visible and NIR regions, respectively. As illustrated in Fig. 4, S14 and S15, the TA spectra without and with BAP show the same characteristic transient bands over the whole visible to NIR region as observed with chemically produced PDI˙^−^. The spectra in the visible region are, however, noisy and slightly distorted near 450 nm, due to the scattering of the continuous excitation light. A prompt decrease in band intensity in both the visible and NIR regions is observed in the presence 1.0 M BAP, keeping the initial concentrations of PDI, TEA, both excitation intensities, and irradiation time constant (Fig. S16). The excited-state lifetime of PDI˙^−^ is not affected by the presence of BAP (Fig. 4b), as observed above with chemically generated PDI˙^−^.

Similar measurements were repeated using fs pump pulses at 410 nm to excite PDI˙^−^. The extinction coefficient of PDI˙^−^ at this wavelength is slightly larger than at 455 nm, the wavelength used in the original study.^7^ As depicted in Fig. S17, the TA spectra contain exclusively features related to the direct excitation of the neutral PDI precursor and to the quenching of its S_1_ state by TEA. No spectral signature of either PDI˙^−^, PDI˙^−^* or any photoproducts can be detected.

#### 2.3.2 Pump–pump–probe measurements in ACN

The inability to achieve photoaccumulation of PDI˙^−^ in ACN is consistent with the absence of free ions upon direct ET quenching of PDI* by TEA or other electron donor like *N*,*N*′-dimethylaniline (DMA) reported previously.^50^ This can be explained by the charge recombination (CR) of the ensuing ion pair, which is ultrafast because of its moderate driving force, corresponding to the barrierless regime in Marcus theory.^51–53^ To overcome this problem, CR of the ion pair was made spin forbidden by undergoing ET quenching of ^3^PDI*. Given that the intrinsic triplet yield of PDI is vanishingly small, ^3^PDI* was populated by triplet CR of the ion pair resulting from the ET quenching of PDI* by iodoanisole (IANI) after excitation of PDI with a 200 ps pulse at 532 nm. As shown previously,^50,54^ CR to the triplet state is no longer spin forbidden in the presence of the heavy atom on the IANI radical cation, and is faster than CR to the singlet neutral ground state, because of its smaller driving force. PDI˙^−^ was then generated upon ET quenching of ^3^PDI* by DMA at low concentrations, to avoid direct quenching of ^1^PDI*. With 10 mM DMA, the highest PDI˙^−^ concentration is reached about 2 µs after excitation of PDI. After this time delay, a TA measurement is performed upon excitation of PDI˙^−^ at either 800 (Vis probing) or 700 nm (NIR probing). In agreement with previous measurents,^50^ the excited-state lifetime of the so-produced PDI˙^−^ amounts to 145 ps, like for chemically or electrochemically produced PDI˙^−^,^20,22^ excluding any hypothetic interference with IANI. As found above in DMF, a prompt decrease in the amplitude of all TA bands is observed in the presence of 1 M BAP (Fig. 4 and S18–S20). In this case again, the decay of PDI˙^−^* is not affected by the addition of BAP, revealing that only static quenching is operative in ACN like in DMF.

## 3 Discussion

### 3.1 The pervasiveness of static quenching in bimolecular photoinduced electron transfer

Although the magnitude of the prompt intensity loss of the TA bands in the presence of BAP varies somehow, between different measurements, due to the difficulty to keep all parameters constant, it is always present and increases systematically with quencher concentration. Therefore, this prompt decrease can unambiguously be attributed to the static quenching of PDI˙^−^*. Although occurrence of static quenching is not surprising in this case, as discussed below, this is an important result as most open-shell ions have generally much shorter excited-state lifetime than PDI˙^−^, a few ps or less.^31–43^ Therefore, their dynamic quenching can be expected to be very inefficient, as noted before.^13,21^

**Fig. 5:**
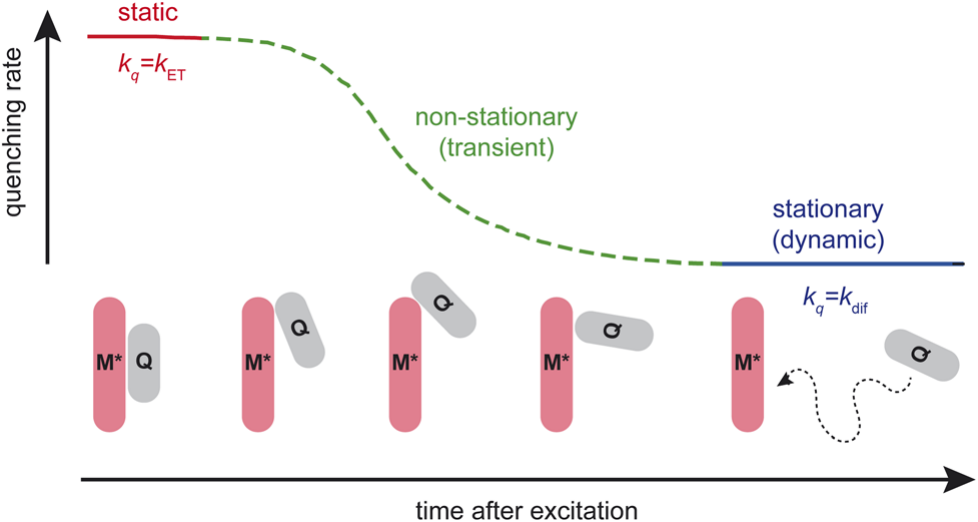
Schematic representation of the three stages of a photoinduced bimolecular electron transfer. Top: time dependence of the quenching rate. Bottom: reactant pairs with decreasing electronic coupling, and undergoing electron transfer at different times after excitation (M*: excited reactant; Q: quencher molecule. Only the diffusive motion of Q is shown for simplicity).

The dynamics of a photoinduced bimolecular ET reaction depend on the spatial distribution of the reactant pairs and three distinct steps can be distinguished (Fig. 5):^26,27,55,56^ (i) in the static stage, ET takes place in the most coupled pairs without diffusion of the reactants. (ii) Once these pairs have reacted, the remaining ones with smaller *V*_ET_ values, because of non-optimal geometry, react more slowly with only minor diffusive motion. This is the non-stationary or transient stage, during which the quenching rate decreases with time as less and less coupled pairs react. (iii) Once all reactant pairs have reacted, new pairs can be generated upon diffusive encounter, and, therefore, quenching becomes diffusion controlled. This latter stationary stage corresponds to what is usually called dynamic quenching. Because of this, the quenching rate is time dependent, decreasing from its largest value, corresponding to the intrinsic ET rate constant, *k*_ET_, to the diffusion rate constant, *k*_dif_.^26,27,55,56^ Static quenching is intrinsic to diffusion-assisted reactions and its probability increases with quencher concentration. Consequently, it does not require any complex formation, pre-association or pre-organisation in the ground state as it is often invoked.^57–60^ These terms, which are often used interchangeably when referring to the static quenching of excited radical ions, presuppose the existence of specific interactions between the reactant pairs. Whereas in a few cases this is supported by the observation of a clear spectral signature,^61^ in most cases, this is inferred from the occurrence of static quenching. Such pre-association can certainly enhance static quenching at lower quencher concentrations, but it is not necessary.

**Fig. 6:**
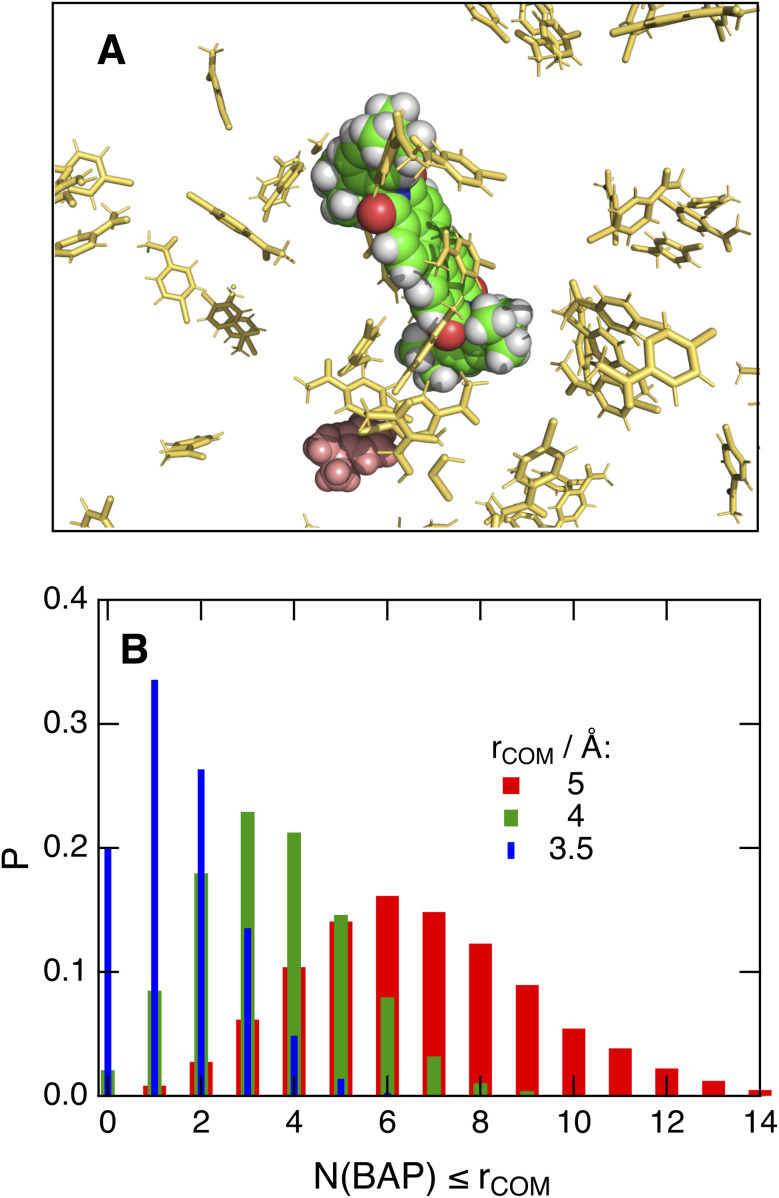
(A) Snapshot from a molecular dynamics simulation of PDI˙^−^ and DMA˙^+^ (red) with 0.9 M BAP (yellow) in DMF (not shown). (B) Histogram of the number of BAP molecules within different centre-of-mass distances, *r*_COM_, from PDI˙^−^.

At the BAP concentrations used here, up to 1 M, the probability to have a PDI˙^−^*−BAP pair with a geometry allowing for a large electronic coupling, should in principle be non-negligible. To confirm this, we performed molecular dynamics simulations of PDI˙^−^ with 0.9 M BAP in DMF, and determined the number of BAP molecules within a given centre-of-mass distance, *r*_COM_, of PDI˙^−^. According to the histogram depicted in Fig. 6, as much as 30% of the anions have a BAP quencher within 3.5 Å, a typical distance for two π-stacked molecules. This figure also shows a simulation snapshot with two BAP molecules π stacked on each side of PDI˙^−^. In the case of the perylene–DMA pair, such geometry was shown to allow for *V*_ET_ values as large as 0.4 eV.^62^ It should be noted that these donor–acceptor stacks could possibly be favoured by dispersion interactions, considering the larger refractive index of BAP (*n*_D_ = 1.57) relatively to DMF (*n*_D_ = 1.43). Given its relatively high concentration, BAP can be considered as a co-solvent and this effect could be viewed as preferential solvation.^63–65^ However, this differs from pre-association as this only involves non-specific interactions.

Such large electronic coupling in π-stacked reactant pairs can explain why only static quenching of PDI˙^−^* is operative with BAP. As mentioned above, the ET driving force for this pair is close to zero, pointing to a quenching rate well below the diffusion limit. However, ET at such unfavourable driving force can still be operative provided *V*_ET_ is sufficiently large.^66–68^ This is clearly the case in the π-stacked pairs, and thus ultrafast ET can take place without any diffusional motion of the reactants. At these large coupling values, ET is an adiabatic reaction,^56,68,69^ and consequently, can no longer be rationalised in terms of Marcus theory,^51^ which is only valid for non-adiabatic processes, with *V*_ET_ values below *k*_B_*T*, about 25 meV at room temperature. Considering that large electronic coupling requires relatively specific mutual orientation of the reactants, most diffusional encounters produce pairs with insufficient *V*_ET_ values for ET to occur. Consequently, dynamic quenching of PDI˙^−^* by BAP is inefficient.

### 3.2 Efficiency of the conPET mechanism with PDI˙^−^ as the photocatalyst

Although our results demonstrate that the short excited-state lifetime of open-shell ions does not prevent their quenching by electron transfer, they evidence other fundamental constraints that could be detrimental to the efficiency of the conPET mechanism as proposed originally.^7^

(i) The conPET mechanism requires the first photoinduced ET process to not only be efficient but to also yield long-lived free ions. For this, charge recombination (CR) of the photoproduced geminate ion pair should be slow enough to allow for the escape to free ions. As shown above, ET quenching of ^1^PDI* by TEA or DMA does not produce free ions in a measurable quantity, because of ultrafast CR, as expected given its moderate driving force. This result might be surprising for TEA, which is known to be a sacrificial donor. However, for TEA to act as a sacrificial donor, its radical cation, TEA˙^+^, should be long-lived enough to react with another TEA molecule.^70,71^ This is certainly not the case neither in ACN nor in DMF using 0.1 M TEA.^50^ The possibility to photoaccumulate PDI˙^−^ in DMF suggests that TEA˙^+^ can react irreversibly before recombining with PDI˙^−^. As TEA˙^+^ is too short lived to react with a TEA molecule at 0.1 M, it most probably undergoes a chemical reaction with the solvent. DMF is, indeed, well known to be more than a solvent.^72–74^ However, as no long-lived PDI˙^−^ population can be detected in the TA experiments,^50^ this reaction appears to be inefficient, with a quantum yield below 1–2%. In principle, this problem could be solved by using the above-described approach, involving first the population of ^3^PDI* with high efficiency and second its ET quenching.^50^

(ii) When using a monochromatic light source, its wavelength should be selected to warrant high absorbance for both photoexcitation steps. Here, it should coincide with an absorption band of both PDI and PDI˙^−^. According to Fig. S2, there is no such wavelength for PDI. Whereas the S_1_ ← S_0_ transition of PDI has significant absorbance at 455 nm, PDI˙^−^ is mostly transparent, as already noted.^21^ Therefore, only a negligibly small PDI˙^−^* population can be expected. This limitation can be easily bypassed using panchromatic illumination.

(iii) Even if a large PDI˙^−^* population could be realised, the quenching product should be long-lived enough for the aryl-halide acceptor anion, A˙^−^, to react further. In other words, its recombination by back ET should be slower that the separation of the PDI A˙^−^ pair. Such condition is not straightforward to achieve when using organic open-shell ions as photocatalysts for several reasons.

(1) Although the molecular orbitals involved in the photoinduced ET and in the ensuing back ET are different, they are generally delocalised over the whole molecules, and therefore, reactant pair geometries with a large *V*_ET_ yield product pairs with large coupling for back ET, *V*_BET_. Because of this, static quenching results automatically in highly coupled product pairs, which, thus, undergo ultrafast back ET.^56,75^

(2) *V*_BET_ can be expected to be significantly smaller for pairs generated upon dynamic quenching. However, this requires using low quencher concentration, ≤ 0.1 M, to avoid static quenching, and a sufficiently long-lived excited state. With a diffusion rate constant of 2 × 10^10^ M^−1^ s^−1^ like in ACN, a quenching efficiency of 0.5 with an acceptor concentration of 0.1 M would require an excited-state lifetime of about 500 ps, significantly longer than that of PDI˙^−^*.

Even if *V*_BET_ were small enough to make back ET a non-adiabatic reaction, slow recombination would still require the process to occur in the Marcus inverted region, *i.e.* to be highly exergonic with −Δ*G*_BET_ > ∼2 eV. Open-shell ions are generally characterised by a small D_1_−D_0_ gap,^76,77^ around 1.3 eV in the case of PDI˙^−^. As a consequence, the driving force for the back ET in the product pairs is even smaller and, thus, this process is ultrafast as occurring in the barrierless regime, *i.e.* with −Δ*G*_BET_ ∼1–1.5 eV, rather than in the inverted region. Alternatively, slow recombination is also expected for back ET in the normal region, with −Δ*G*_BET_ ≤ 0.6–0.7 eV.^30^ However, this last approach requires using a strong electron acceptor and, consequently, a large fraction of the energy of the excited photocatalyst is lost during the highly exergonic quenching step. This would significantly limit the advantage of the conPET mechanism compared to the more standard one-photon redox catalysis.

(3) Charge recombination of an ion pair can be made spin-forbidden and, thus, slow, by quenching the triplet state. Contrary to closed-shell molecules with the first triplet excited state below S_1_, the lowest quartet state of a radical ion, Q_1_, is at significantly higher energy than the lowest doublet excited state, D_1_, as it is associated with a two-electron excitation.^78^ Consequently, the quenching product, PDI+A˙^−^, is also in a doublet state and its recombination to PDI+A˙^−^ is spin allowed. Making back ET spin-forbidden by quenching Q_1_ instead of D_1_ is not feasible because this state cannot be photopopulated from D_0_.^78^

The above discussion reveals several shortcomings, which limit the efficiency of the conPET mechanism when using PDI˙^−^ as the photocatalyst. Indeed, the quantum efficiency of each ET step is very low because of ultrafast recombination and therefore only a vanishingly small overall efficiency can be expected. However, this does not imply that this conPET approach is not operative. Indeed, upon long enough photo-illumination, processes with very low quantum yield can still take place. Similar problems can be anticipated with other open-shell anions, unless their excited state is long-lived enough to use low acceptor concentrations and prevent static quenching.

Consequently, the exact mechanism responsible for the transformation of BAP to AP upon illumination of PDI cannot be unambiguously determined. However, our results suggest that DMF could play a significant role, as it is not as inert a solvent as ACN, where no photoaccumulation of PDI˙^−^ takes place (Fig. S2). Considering that the formation of AP was found to still occur once all PDI˙^−^ species are consumed,^21^ a possible mechanism could involve a reaction between BAP and a photodegradation product of PDI˙^−^ in DMF.

## 4 Conclusions

Our results reveal that static electron transfer quenching is an efficient reaction pathway of PDI˙^−^*, which can be operative even in the absence of significant dynamic quenching. Static quenching is intrinsic to diffusion-assisted photoredox reactions and does not require any preorganisation of the reactants in the ground state, although this process can certainly be beneficial at low quencher concentrations. However, static quenching occurs with highly-coupled reactant pairs and yield highly-coupled product pairs, which undergo rapid recombination by back electron transfer, before any ‘useful’ follow-up reaction can take place. In principle, less coupled pairs could be produced by dynamic quenching, provided that the excited-state lifetime of the radical ion is long-lived enough. However, recombination might still be very fast because of its moderate driving force, which makes electron transfer a barrierless process according to Marcus theory. These factors strongly limit the performance of PDI˙^−^ as a photoredox catalyst, independently on whether it is produced electro- or photochemically. Similar problems can be expected for other radical anions with a short-lived excited state and for which static quenching is predominant.

In practice, a low efficiency implies that long irradiation times are required for the desired reaction to take place. Whilst this might not be a problem *per se*, it increases the probability of other unwanted side reactions to occur.

## Author contributions

ESR and DHCN designed and performed the spectroscopic experiments and the analysis. EV conceived the project and carried out the molecular dynamics simulations. ESR wrote the initial manuscript. The final version was written by EV with the help of all authors.

## Conflicts of interest

There are no conflicts to declare.

## Supplementary Material

SC-017-D6SC02462A-s001

## Data Availability

All data can be downloaded from https://doi.org/10.26037/yareta:iavqletmovcpplucnrsjkryrm4. Supplementary information (SI): experimental methods, stationary absorption spectra, additional transient electronic absorption spectra and global analysis. See DOI: https://doi.org/10.1039/d6sc02462a.
