# Supplementary material for: Evidence of static quenching in the photoredox activity of perylene diimide radical anions: implications for consecutive photoinduced electron transfer photocatalysis
Source: Chem Sci. 2026 May 22;17(28):13917–25. doi: 10.1039/d6sc02462a (PMC13248644; doi:10.1039/d6sc02462a)
Supplement: SC-017-D6SC02462A-s001 [file SC-017-D6SC02462A-s001.pdf]

Supplementary Information (SI) for Chemical Science.
This journal is © The Royal Society of Chemistry 2026

Evidence of static quenching in the photoredox activity of perylene diimide radical anion: Implications for consecutive photoinduced electron transfer photocatalysis

Estefanía Sucre-Rosales, Daniel H. Cruz Neto, and Eric Vauthey*

Department of Physical Chemistry, University of Geneva, 30 Quai Ernest-Ansermet, CH-1211 Geneva 4, Switzerland. E-mail: eric.vauthey@unige.ch

Contents

S1 Experimental Methods **4**
S1.1 Solvents and reactants . . . 4
S1.2 Chemical generation of $PDI^{\bullet -}$ in DMF and ACN . . . 4
S1.3 Stationary electronic absorption . . . 4
S1.4 Stationary fluorescence . . . 4
S1.5 Transient absorption spectroscopy . . . 4
S1.5.1 Pump-probe measurements . . . 4
S1.5.2 Pump-probe measurements with photoaccumulated $PDI^{\bullet -}$ . . . 5
S1.5.3 Pump-pump-probe measurements . . . 5
S1.6 Molecular dynamics simulations . . . 5

S2 Additional results **6**
S2.1 Stationary electronic absorption . . . 6
S2.2 Transient absorption of chemically-generated $PDI^{\bullet -}$ . . . 8
S2.3 Pump-probe measurements with photoaccumulated $PDI^{\bullet -}$ . . . 14
S2.4 Pump-pump-probe measurements . . . 16
S2.5 Comparing the quenching of $PDI^{\bullet -*}$ by BPA under different experimental conditions . . . 18

List of Figures

S1 Absorption spectra of chemically generated $PDI^{\bullet -}$ in DMF in the presence of increasing amounts 4-nitrobenzaldehyde (NBZ) in DMF 6
S2 Absorption spectra recorded after different irradiation times (in min.) with a high-repetition rate ns pulsed laser diode at 450 nm of a solution containing PDI, TEA (0.1 M), and 4-bromoacetophenone (BAP) (1 M) in DMF. 7
S3 Same as Figure S2 but in ACN. 7
S4 Left: transient absorption spectra measured upon 800 nm excitation of $PDI^{\bullet -}$ in DMF, with increasing amounts of 4-bromo acetophenone (BAP). Right: evolution-associated spectra and time constants obtained from global analysis assuming a series of two successive exponential steps. Note the decrease of the initial band intensity upon increasing BAP concentration. 8
S5 Same as Figure S4 but upon 960 nm excitation 9
S6 Same as Figure S4 but upon 960 nm excitation and the NIR probing. 10
S7 Same as Figure S4 but in ACN and upon 700 nm excitation. 10
S8 Same as Figure S4 but using nitrobenzene (NBZ) as quencher. 11
S9 Stern-Volmer plot for the quenching of $PDI^{\bullet -*}$ by nitrobenzene in DMF. The lifetime ratio was calculated with the second time constant (B$\rightarrow$) obtained from the global analysis of the data measured with 800 nm excitation. The resulting quenching rate constant, $k_q = 1.8 \times 10^{10}\,\mathrm{M^{-1}s^{-1}}$, is comparable to that reported in literature ($k_q = 1.1 \times 10^{10}\,\mathrm{M^{-1}s^{-1}}$).[1] 11
S10 Same as Figure S8 but upon 960 nm excitation. 12
S11 Same as Figure S9 but using the data recorded upon 960 nm excitation. The resulting quenching rate constant, $k_q = 1.0 \times 10^{10}\,\mathrm{M^{-1}s^{-1}}$, is comparable to that reported in literature ($k_q = 1.1 \times 10^{10}\,\mathrm{M^{-1}s^{-1}}$).[1] 13
S12 Same as Figure S4 but with 4-nitrobenzaldehyde (NBA). 13
S13 Transient absorption spectra recorded after 960 nm excitation of a 1 M solution of BAP in DMF. 13
S14 Left: transient absorption spectra recorded at different time delays after 800 nm excitation of photoaccumulated $PDI^{\bullet -}$ without and with BAP in DMF. $PDI^{\bullet -}$ is generated by continuous irradiation of PDI at 450 in the presence of 0.1 M TEA. Right: evolution-associated difference absorption spectra and time constants obtained upon global analysis of the data assuming a single exponential step. 14
S15 Same as Figure S14 but with 700 nm excitation and NIR probing. 14
S16 Time profiles (top) and intensity-normalised time profiles (bottom) extracted from the data shown in Figure S14 (465 nm) and S15 (960 nm). 15
S17 Same as Figure S14 but with the pump pulses at 410 nm. 15

S18 Left: pump-pump-probe transient absorption spectra measured with different BAP concentrations at various time s after 800 nm excitation of PDI$^{\bullet-}$ in deaerated acetonitrile, 2μs after its generation by pulsed excitation at 532 nm of PDI with 1 M iodoanisole and 10 mM N,N-dimethylaniline. Right: evolution-associated difference absorption spectra and time constants obtained from global analysis of the data assuming a single exponential step. 16
S19 Same as Figure S18 but with 700 nm excitation and NIR probing. 17
S20 Time profiles (top) and intensity-normalised time profiles (bottom) extracted from the data shown in Figure S18 (465 nm) and S19 (960 nm). 17
S21 Intensity ratio of the PDI$^{\bullet-*}$ band at 460 nm measured 10 ps after excitation without and with different BAP concentrations. 18

S1 Experimental Methods

S1.1 Solvents and reactants

All solvents were of HPLC and/or spectroscopic grade, and were used as received. The commercial PDI derivative used was used as received.

S1.2 Chemical generation of $PDI^{\bullet -}$ in DMF and ACN

The reduction reaction was performed as reported in ref. 1 . In brief, equimolar quantities of PDI and tetrakis(dimethylamino)ethylene (TDAE) were added to a vial inside a glovebox under nitrogen atmosphere, with the corresponding amount of DMF/ACN and quencher, to ensure that all solutions were prepared with the same concentration of $PDI^{\bullet -}$. The solutions were added then to fill up completely a 1 mm cuvette with a teflon lined screw stopper that was sealed thrice with parafilm, and measured immediately.

S1.3 Stationary electronic absorption

All samples were measured in 1 cm or 1 mm cuvettes. The stationary electronic absorption spectra were measured using a Cary 50 spectrometer.

S1.4 Stationary fluorescence

The fluorescence from chemically generated $PDI^{\bullet -}$ was measured in 1 mm cuvettes using a Horiba NanoLog fluorometer with an NIR detector (R-5509-73).

S1.5 Transient absorption spectroscopy

S1.5.1 Pump-probe measurements

Ultrafast electronic transient absorption (TA) measurements were performed with a setup described in ref. 2,3 and based on an amplified Ti:Sapphire system (Solstice Ace, Spectra-Physics), producing 35 fs pulses centred at 800 nm with a 5 kHz repetition rate. For measurement with pump pulses at 800 nm, a fraction of the amplifier output was used. Pump pulses at 700 and 960 nm were generated using a TOPAS-Prime combined with a NirUVis module (Light Conversion). The pump irradiance on the sample was between 0.15 and 0.75 $mJ \cdot cm^{-2}$. Probing was achieved from using white light pulses generated in either a 3 mm CaF_2 plate (320 to 750 nm)[3] or a 3 mm YAG plate (NIR).[2] The probe pulses were then split into sample and reference pulses. Both pump and probe pulses were focused and overlapped onto the sample (100 and 60 μm diameter, respectively). The polarisation of the pump pulses was at magic angle with respect to that of the probe pulses. The transient absorption signal was checked prior to the measurement to scale linearly with the pump irradiance. The sample cell was 1 mm thick and the IRF had a FWHM varying between 80 and 350 fs, depending on the probe wavelength. All samples were kept under nitrogen flow during the entire duration of the measurement (around 1.5-2 h), or prepared under nitrogen in a glovebox.

For the data treatment, the pixel-to-wavelength conversion was done using a standard filter of Holmium oxide, which shows narrow bands in the UV-Vis spectral region. Global analysis was performed using a Matlab script.[4]

S1.5.2 Pump-probe measurements with photoaccumulated $PDI^{\bullet-}$

The transient absorption measurement were performed with the same setup as described above using 800 nm pump pulses with the only difference that the sample was continuously irradiated at 450 nm with a high-repetition rate (10 MHz) ns pulse laser diode (Thorlab, NPL45B).

S1.5.3 Pump-pump-probe measurements

The pump-pump-probe measurements were carried out by introducing an additional pump pulse at 532 nm in the above-described transient absorption setup, as described in detail elsewhere.[5] The pump pulses at 532 nm were produced by a passively Q-switched, frequency doubled Nd:YAG laser (Teem Photonics, Powerchip NanoUV). These pulses were at a repetition rate of 500 Hz, with an energy of approximately 20 μJ, and a duration of 300 ps. The second pump pulse was at 800 nm and was delayed by 2 μs with respect to the first one using a high precision time delay counter (Stanford Research Systems, SR620).

S1.6 Molecular dynamics simulations

Molecular dynamics (MD) simulations were carried out using GROMACS 2023.1.[6] The optimised structures of $PDI^{\bullet-}$, $DMA^{\bullet+}$ and BAP were determined from quantum-chemical calculations in the gas phase at the DFT level (B3LYP/6-31G(d,p)) using Gaussian 16.[7] The topologies for $PDI^{\bullet-}$, $DMA^{\bullet+}$ and BAP were generated using the Antechamber Python parser interface (ACPYPE),[8] with the general Amber force field (GAFF).[9] The atomic charges were determined from CHELPG fits of the electrostatic potential obtained from the quantum-chemical calculations.[10] The GAFF-ESP-2018 force field was used for the solvents.[11] A periodic cubic box (5x5x5 nm^3) was used for the simulations, which were performed at constant pressure (1 atm) and temperature (295 K) with 2 fs steps for 50 ns.

Non-bonded interactions were evaluated with a cutoff of 1.2 nm, and long-range electrostatic interactions were accounted for by the particle mesh Ewald method,[12] with 0.16 nm grid spacing and forth-order interpolation. A long-range dispersion correction for energy was also included. The LINCS algorithm[13] was used to constrain the bonds of all system components. The equilibration of the system was ensured by inspecting the total energy drift. The isothermal-isobaric ensemble, NPT, was used for all productions with the Noose-Hoover thermostat at 295 K,[14] and the c-rescale barostat[15] at 1 atm using coupling constants 0.5 and 5 ps respectively.

S2 Additional results

S2.1 Stationary electronic absorption

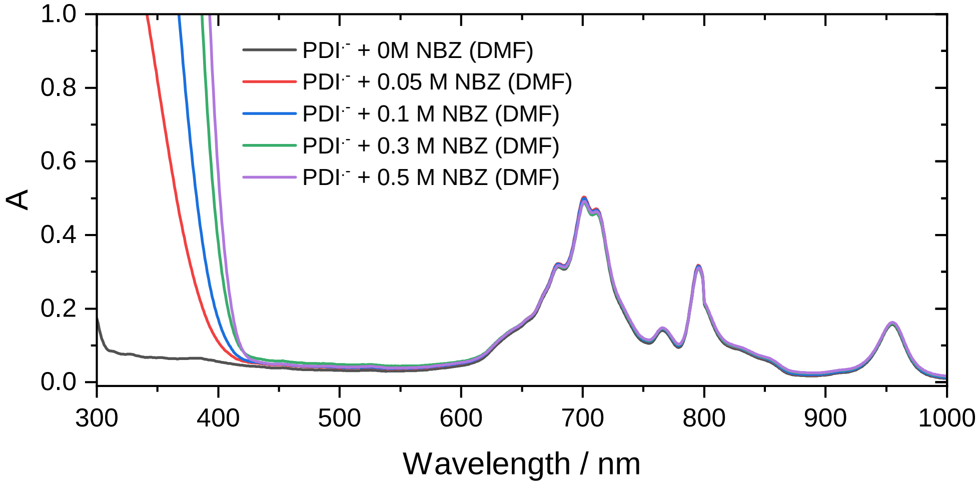

Figure S1 Absorption spectra of chemically generated $PDI^{\bullet-}$ in DMF in the presence of increasing amounts 4-nitrobenzaldehyde (NBZ) in DMF

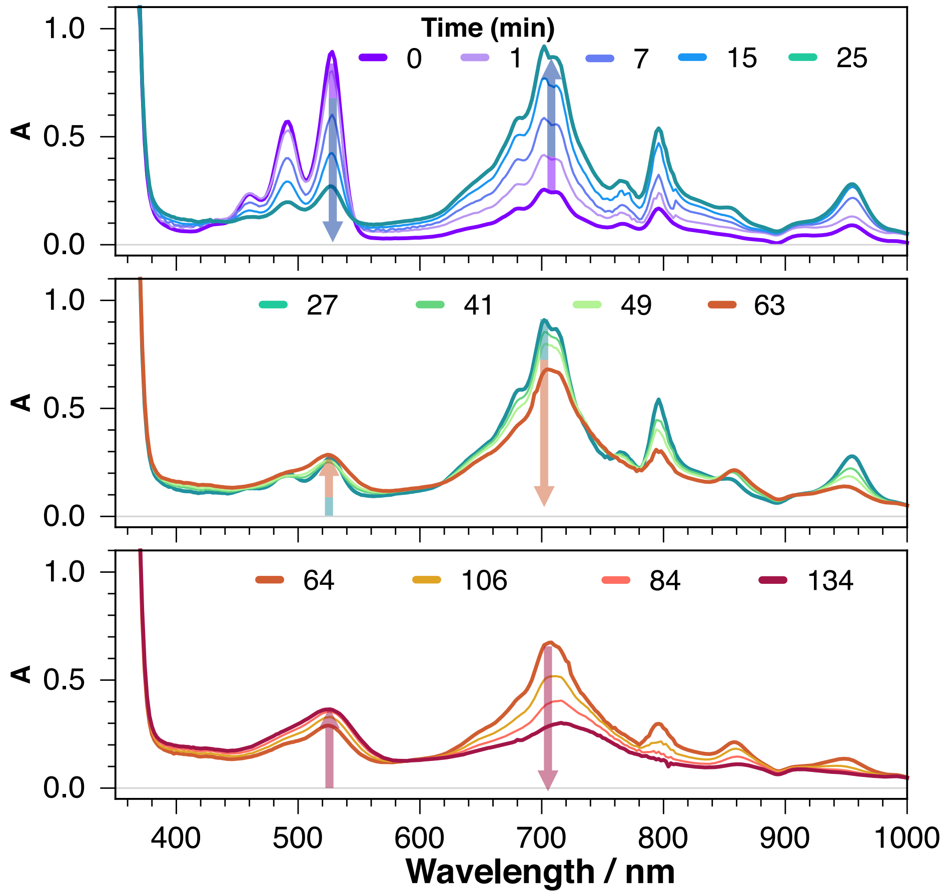

Figure S2 Absorption spectra recorded after different irradiation times (in min.) with a high-repetition rate ns pulsed laser diode at 450 nm of a solution containing PDI, TEA (0.1 M), and 4-bromoacetophenone (BAP) (1 M) in DMF.

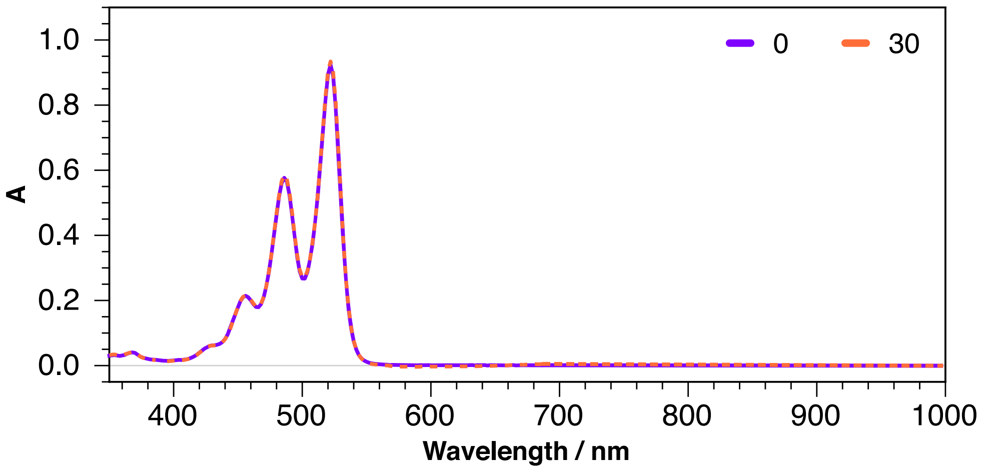

Figure S3 Same as Figure S2 but in ACN.

S2.2 Transient absorption of chemically-generated PDI$^{\bullet-}$

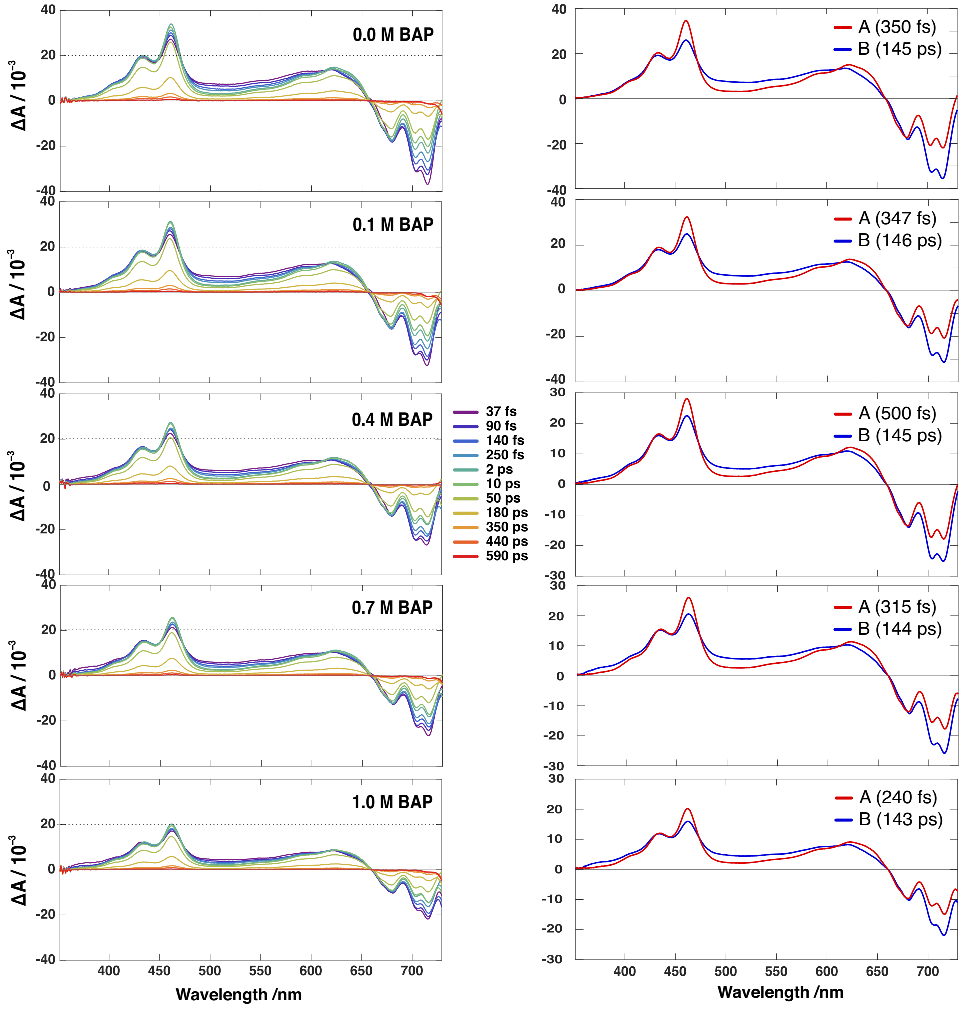

Figure S4 Left: transient absorption spectra measured upon 800 nm excitation of PDI$^{\bullet-}$ in DMF, with increasing amounts of 4-bromo acetophenone (BAP). Right: evolution-associated spectra and time constants obtained from global analysis assuming a series of two successive exponential steps. Note the decrease of the initial band intensity upon increasing BAP concentration.

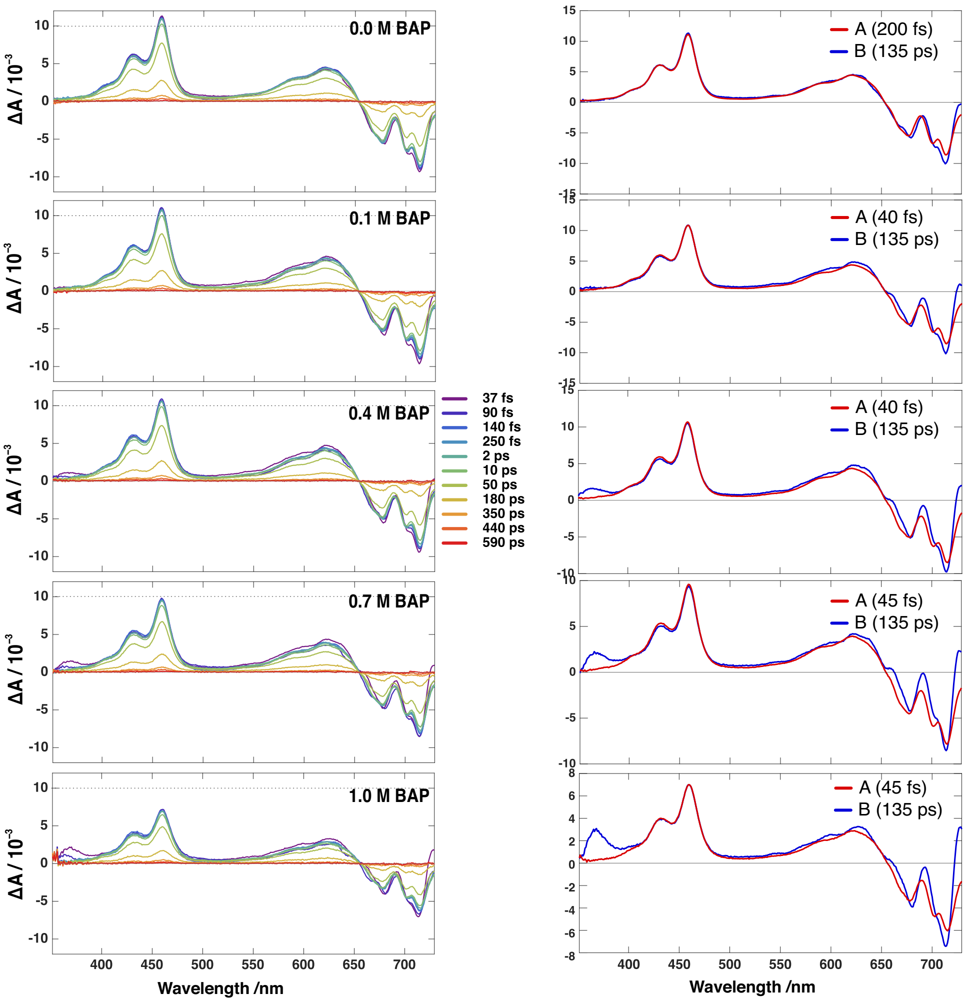

Figure S5 Same as Figure S4 but upon 960 nm excitation

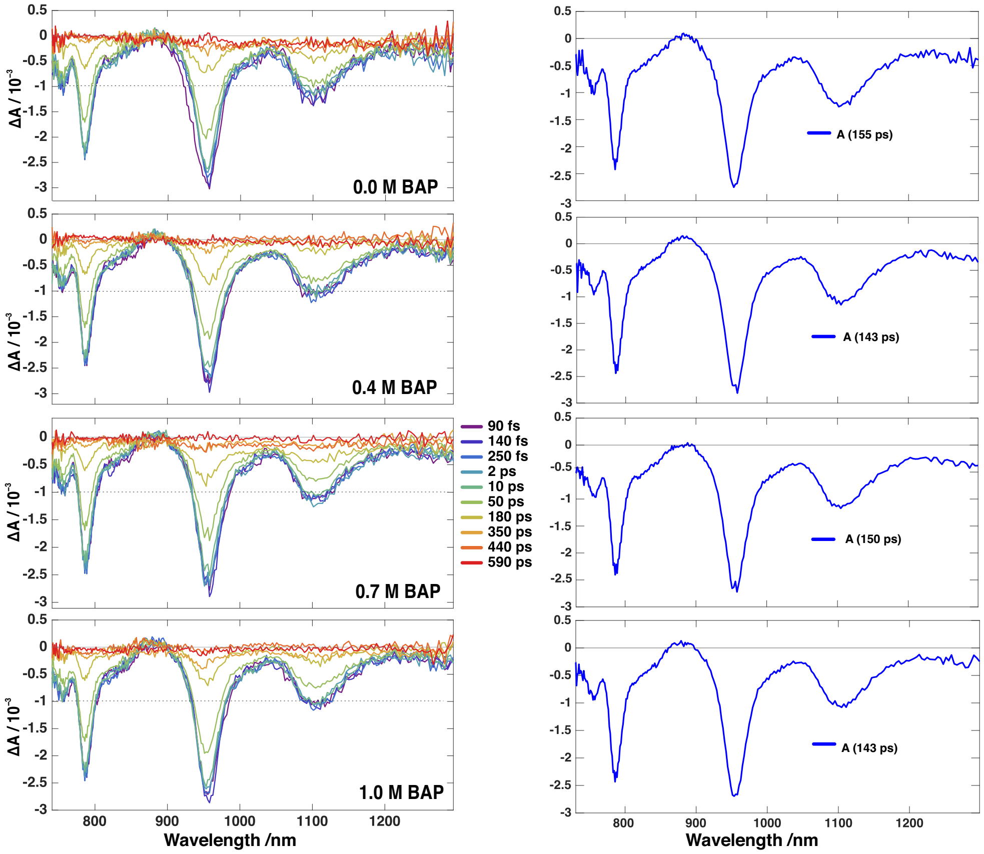

Figure S6 Same as Figure S4 but upon 960 nm excitation and the NIR probing.

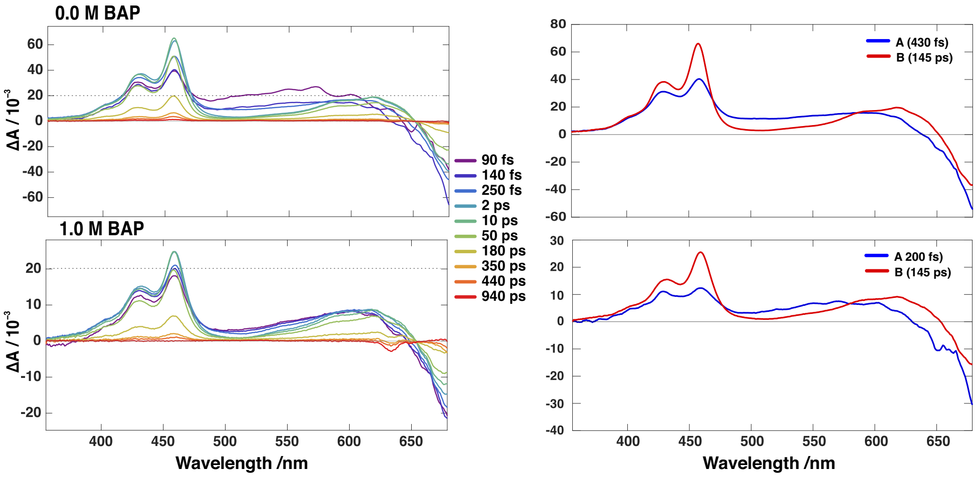

Figure S7 Same as Figure S4 but in ACN and upon 700 nm excitation.

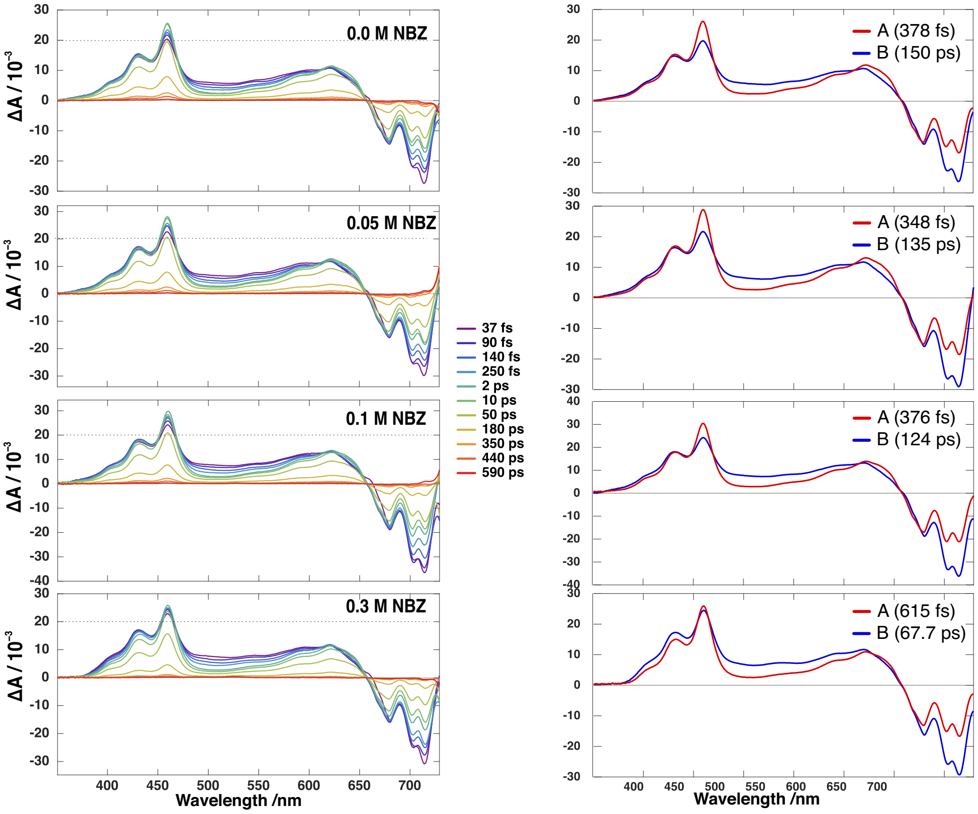

Figure S8 Same as Figure S4 but using nitrobenzene (NBZ) as quencher.

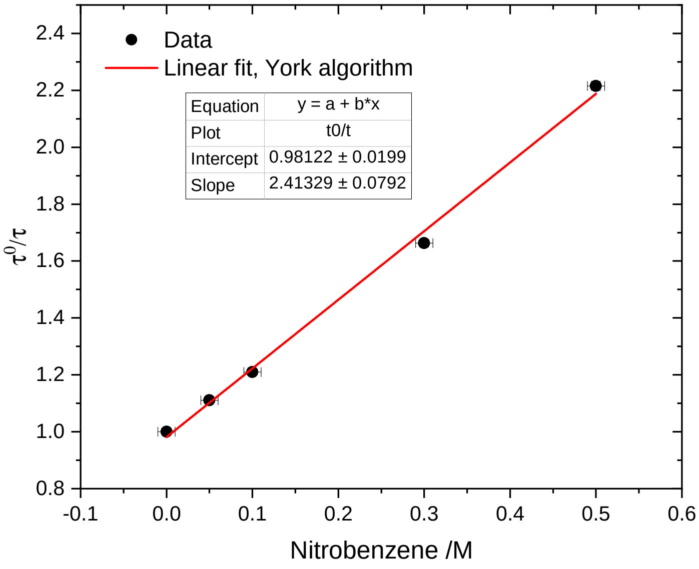

Figure S9 Stern-Volmer plot for the quenching of $PDI^{\bullet-*}$ by nitrobenzene in DMF. The lifetime ratio was calculated with the second time constant (B→) obtained from the global analysis of the data measured with 800 nm excitation. The resulting quenching rate constant, $k_q = 1.8 \times 10^{10}\,\mathrm{M}^{-1}\mathrm{s}^{-1}$, is comparable to that reported in literature ($k_q = 1.1 \times 10^{10}\,\mathrm{M}^{-1}\mathrm{s}^{-1}$).[1]

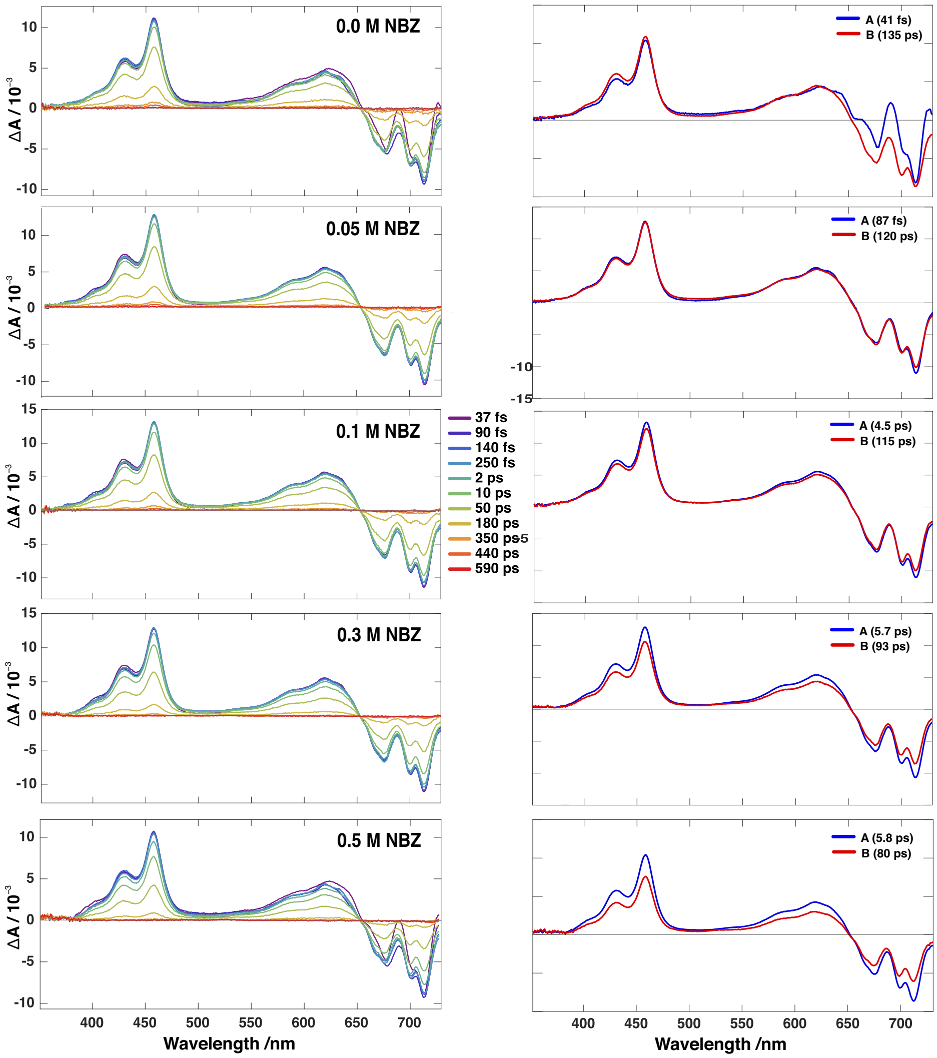

Figure S10 Same as Figure S8 but upon 960 nm excitation.

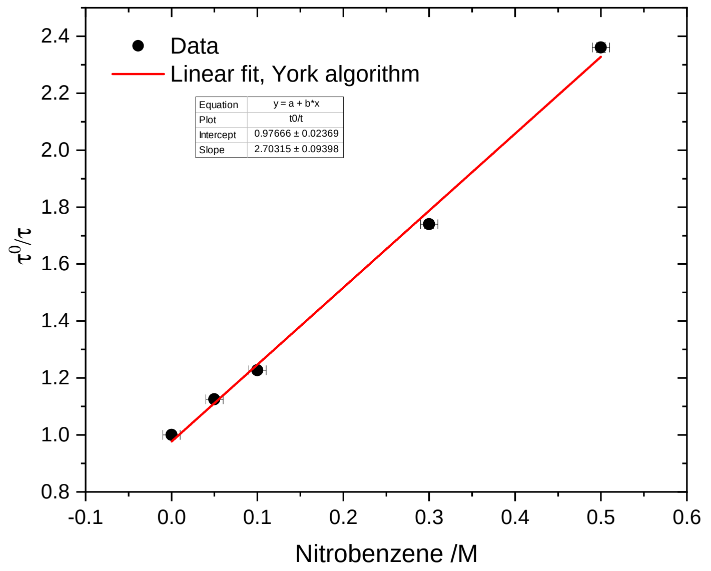

Figure S11 Same as Figure S9 but using the data recorded upon 960 nm excitation. The resulting quenching rate constant, $k_q = 1.0 \times 10^{10}\,\mathrm{M}^{-1}\mathrm{s}^{-1}$, is comparable to that reported in literature ($k_q = 1.1 \times 10^{10}\,\mathrm{M}^{-1}\mathrm{s}^{-1}$).[1]

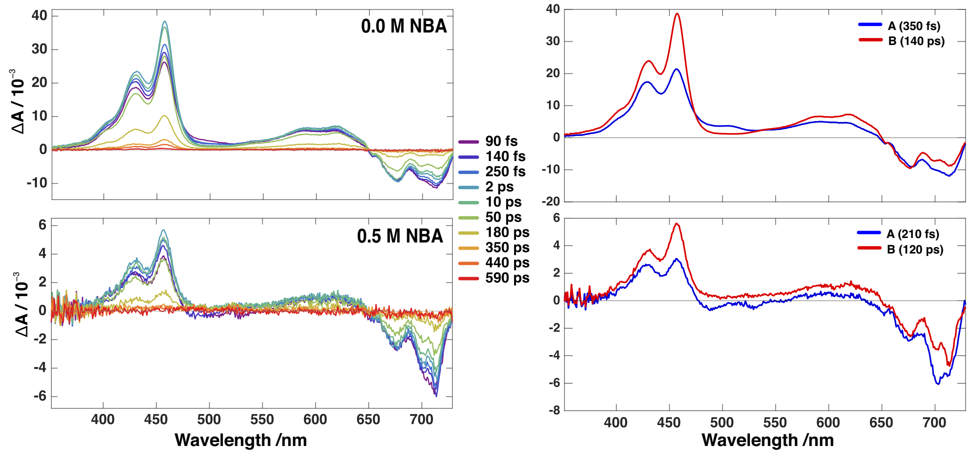

Figure S12 Same as Figure S4 but with 4-nitrobenzaldehyde (NBA).

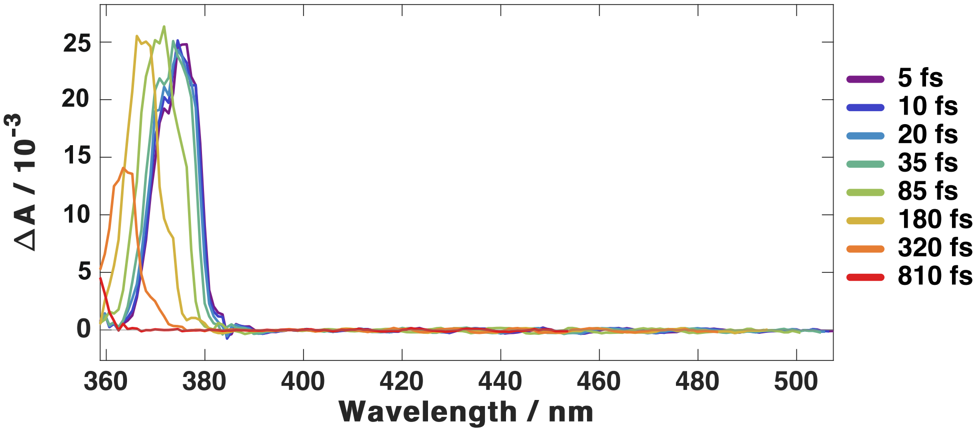

Figure S13 Transient absorption spectra recorded after 960 nm excitation of a 1 M solution of BAP in DMF.

S2.3 Pump-probe measurements with photoaccumulated PDI$^{\bullet-}$

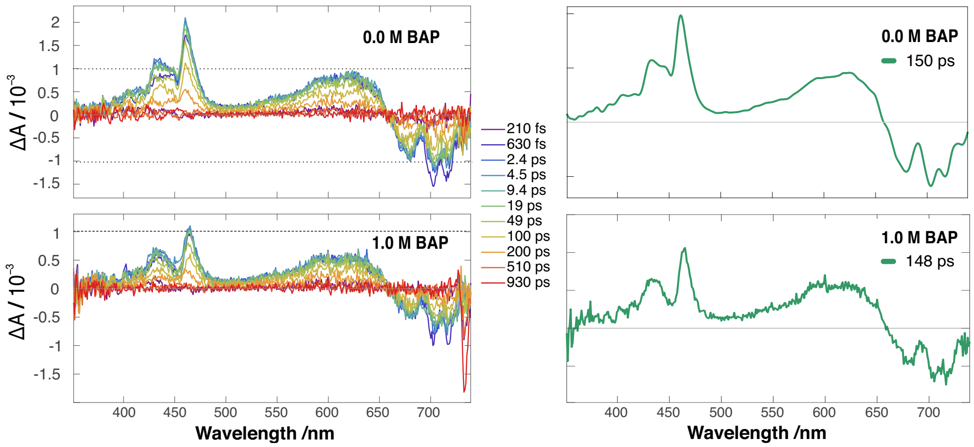

Figure S14 Left: transient absorption spectra recorded at different time delays after 800 nm excitation of photoaccumulated PDI$^{\bullet-}$ without and with BAP in DMF. PDI$^{\bullet-}$ is generated by continuous irradiation of PDI at 450 in the presence of 0.1 M TEA. Right: evolution-associated difference absorption spectra and time constants obtained upon global analysis of the data assuming a single exponential step.

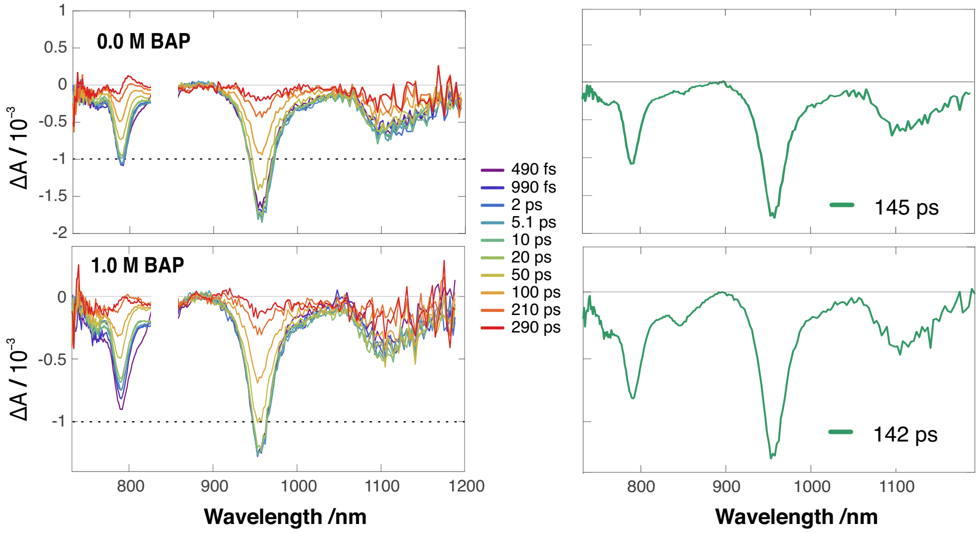

Figure S15 Same as Figure S14 but with 700 nm excitation and NIR probing.

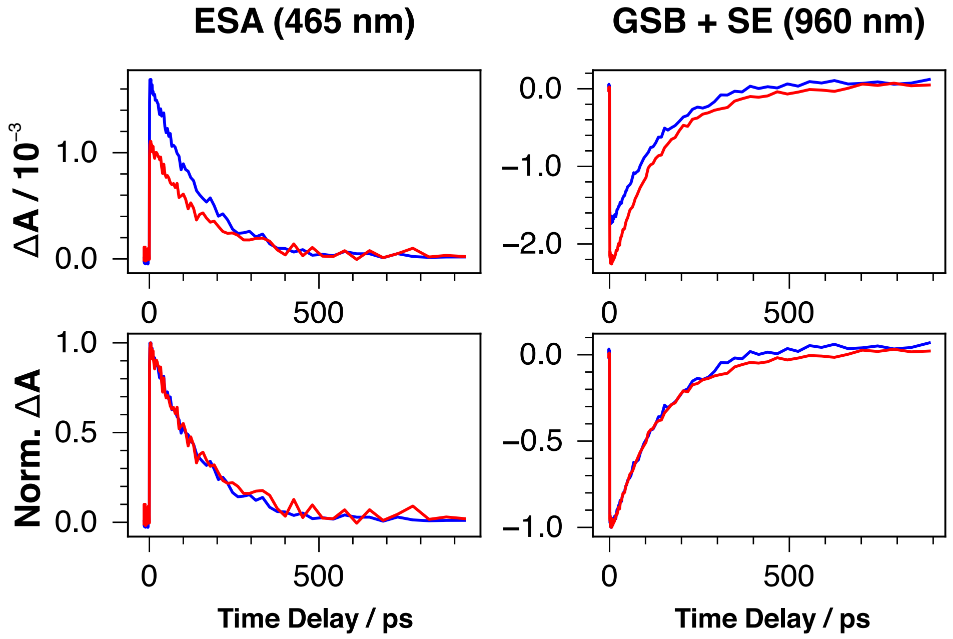

Figure S16 Time profiles (top) and intensity-normalised time profiles (bottom) extracted from the data shown in Figure S14 (465 nm) and S15 (960 nm).

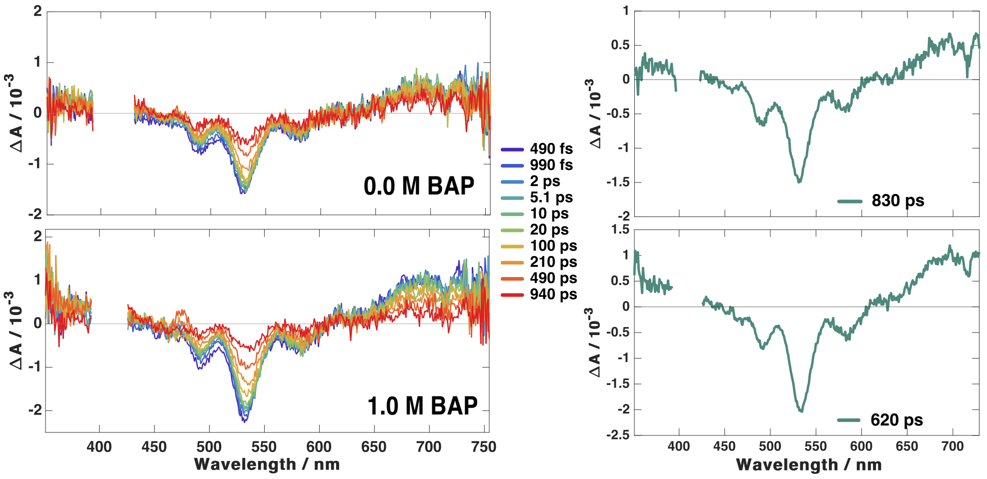

Figure S17 Same as Figure S14 but with the pump pulses at 410 nm.

S2.4 Pump-pump-probe measurements

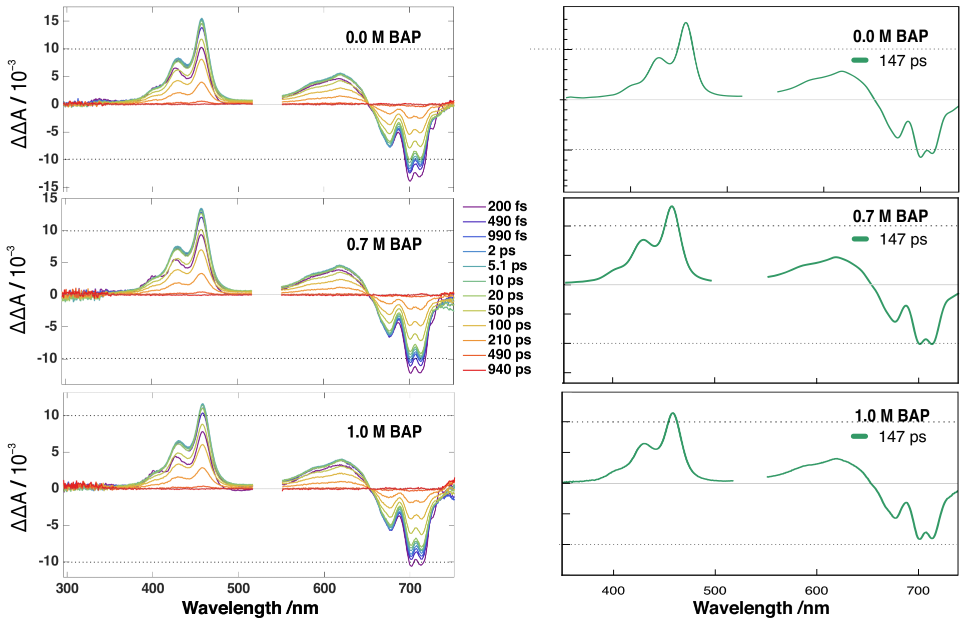

Figure S18 Left: pump-pump-probe transient absorption spectra measured with different BAP concentrations at various time s after 800 nm excitation of $PDI^{\bullet-}$ in deaerated acetonitrile, 2μs after its generation by pulsed excitation at 532 nm of PDI with 1 M iodoanisole and 10 mM N,N-dimethylaniline. Right: evolution-associated difference absorption spectra and time constants obtained from global analysis of the data assuming a single exponential step.

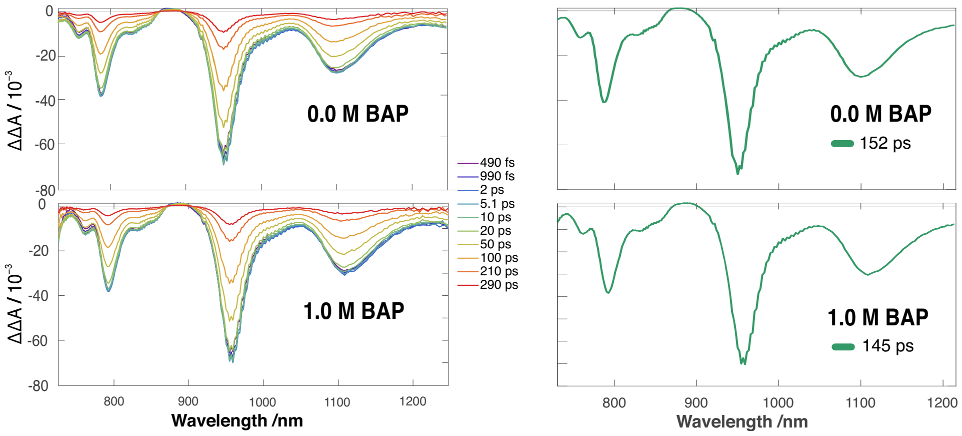

Figure S19 Same as Figure S18 but with 700 nm excitation and NIR probing.

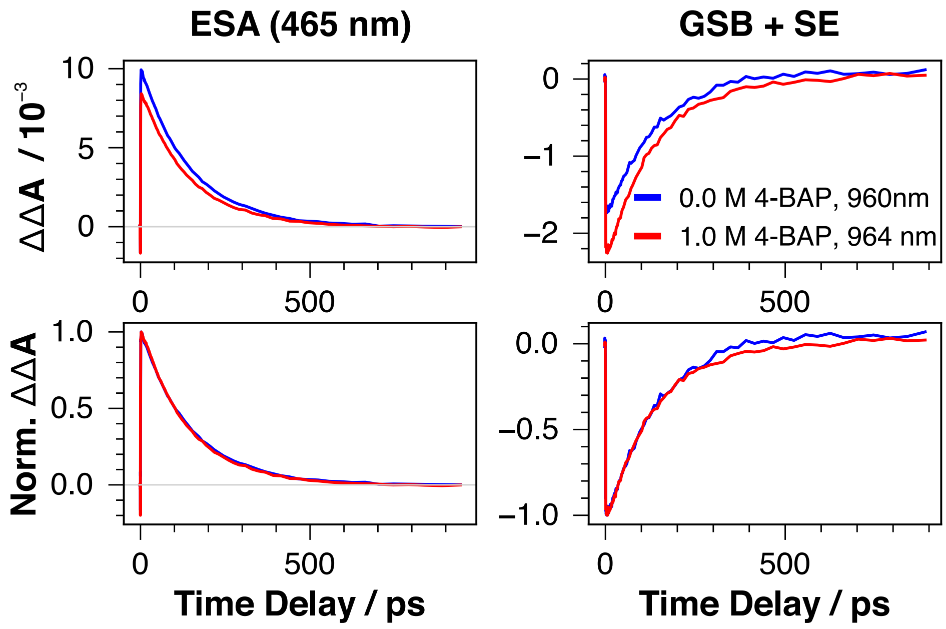

Figure S20 Time profiles (top) and intensity-normalised time profiles (bottom) extracted from the data shown in Figure S18 (465 nm) and S19 (960 nm).

S2.5 Comparing the quenching of PDI$^{\bullet-*}$ by BPA under different experimental conditions

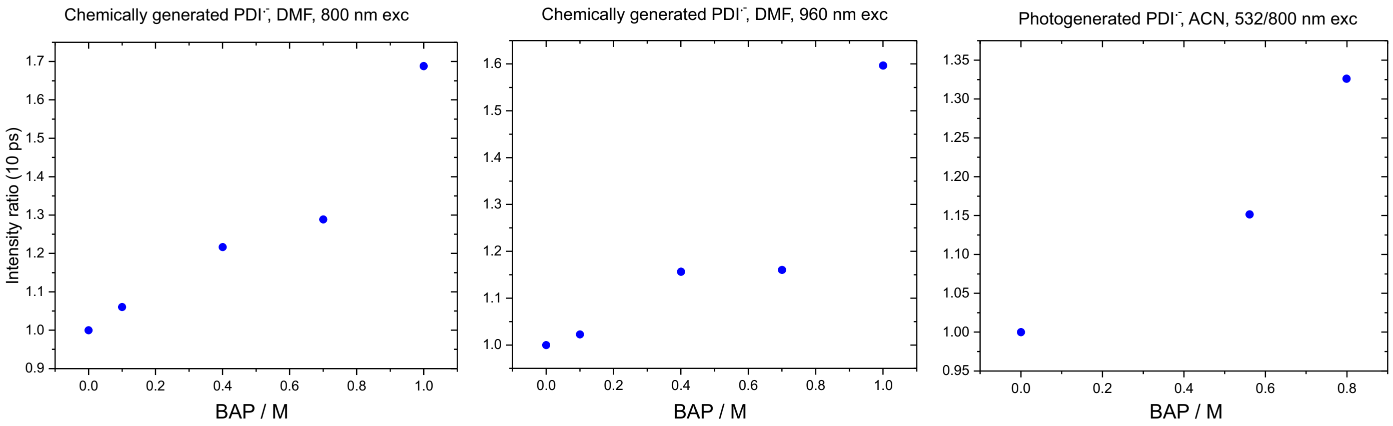

Figure S21 Intensity ratio of the PDI$^{\bullet-*}$ band at 460 nm measured 10 ps after excitation without and with different BAP concentrations.

References

[1] C. J. Zeman, S. Kim, F. Zhang, K. S. Schanze, *J. Am. Chem. Soc.* **2020**, *142*, 2204.

[2] A. Aster, F. Zinna, C. Rumble, J. Lacour, E. Vauthey, *J. Am. Chem. Soc.* **2021**, *143*, 2361.

[3] J. S. Beckwith, A. Aster, E. Vauthey, *Phys. Chem. Chem. Phys.* **2022**, *24*, 568.

[4] R. J. Fernández-Terán, E. Sucre-Rosales, L. Echevarria, F. E. Hernández, *J. Chem. Educ.* **2022**, *99*, 2327.

[5] D. H. Cruz Neto, E. Sucre-Rosales, E. Vauthey, *J. Phys. Chem. Lett.* **2025**, *16*, 13241.

[6] M. J. Abraham, T. Murtola, R. Schulz, S. Páll, J. C. Smith, B. Hess, E. Lindahl, *SoftwareX* **2015**, *1-2*, 19.

[7] M. J. Frisch, G. W. Trucks, H. B. Schlegel, G. E. Scuseria, M. A. Robb, J. R. Cheeseman, G. Scalmani, V. Barone, G. A. Petersson, H. Nakatsuji, X. Li, M. Caricato, A. V. Marenich, J. Bloino, B. G. Janesko, R. Gomperts, B. Mennucci, H. P. Hratchian, J. V. Ortiz, A. F. Izmaylov, J. L. Sonnenberg, D. Williams-Young, F. Ding, F. Lipparini, F. Egidi, J. Goings, B. Peng, A. Petrone, T. Henderson, D. Ranasinghe, V. G. Zakrzewski, J. Gao, N. Rega, G. Zheng, W. Liang, M. Hada, M. Ehara, K. Toyota, R. Fukuda, J. Hasegawa, M. Ishida, T. Nakajima, Y. Honda, O. Kitao, H. Nakai, T. Vreven, K. Throssell, J. A. Montgomery Jr., J. E. Peralta, F. Ogliaro, M. J. Bearpark, J. J. Heyd, E. N. Brothers, K. N. Kudin, V. N. Staroverov, T. A. Keith, R. Kobayashi, J. Normand, K. Raghavachari, A. P. Rendell, J. C. Burant, S. S. Iyengar, J. Tomasi, M. Cossi, J. M. Millam, M. Klene, C. Adamo, R. Cammi, J. W. Ochterski, R. L. Martin, K. Morokuma, O. Farkas, J. B. Foresman, D. J. Fox **2016**.

[8] A. W. Sousa da Silva, W. F. Vranken, *BMC Res. Notes* **2012**, *5*, 367.

[9] J. Wang, R. M. Wolf, J. W. Caldwell, P. A. Kollman, D. A. Case, *J. Comput. Chem.* **2004**, *25*, 1157.

[10] L. E. Chirlian, M. M. Francl, *J. Comput. Chem.* **1987**, *8*, 894.

[11] D. van der Spoel, M. M. Ghahremanpour, J. A. Lemkul, *J. Phys. Chem. A* **2018**, *122*, 8982.

[12] T. Darden, D. York, L. Pedersen, *J. Chem. Phys.* **1993**, *98*, 10089.

[13] B. Hess, H. Bekker, H. J. C. Berendsen, J. G. E. M. Fraaije, *J. Comput. Chem.* **1997**, *18*, 1463.

[14] W. G. Hoover, *Phys. Rev. A* **1985**, *31*, 1695.

[15] M. Bernetti, G. Bussi, *J. Chem. Phys.* **2020**, *153*, 114107.
